# Elemental Substitution at Tl Site of Tl_1−*x*_X*_x_*(Ba, Sr)CaCu_2_O_7_ Superconductor with X = Cr, Bi, Pb, Se, and Te

**DOI:** 10.3390/ma16114022

**Published:** 2023-05-27

**Authors:** Jaafar Nur-Akasyah, Roslan Abd-Shukor, Tet Vui Chong

**Affiliations:** 1 Faculty of Engineering and Quantity Surveying, INTI International University, Persiaran Perdana BBN, Putra Nilai, Nilai 71800, Negeri Sembilan, Malaysia; 2 Department of Applied Physics, Universiti Kebangsaan Malaysia, Bangi 43600, Selangor, Malaysia

**Keywords:** Tl(Ba,Sr)CaCu_2_O_7_, superconductivity, Tl-1212

## Abstract

The effects of elemental substitutions at the Tl site of a Tl_1−*x*_X*_x_*(Ba, Sr)CaCu_2_O_7_ superconductor with X = Cr, Bi, Pb, Se, and Te were investigated. This study aimed to determine the elements that enhance and suppress the superconducting transition temperature of the Tl_1−*x*_X*_x_*(Ba, Sr)CaCu_2_O_7_ (Tl-1212) phase. The selected elements belong to the groups of transition metal, post-transition metal, non-metal, and metalloid. The relationship between the transition temperature and ionic radius of the elements was also discussed. The samples were prepared by the solid-state reaction method. The XRD patterns showed a single Tl-1212 phase was formed in the non- and Cr-substituted (*x* = 0.15) samples. The Cr-substituted samples (*x* = 0.4) showed a plate-like structure with smaller voids. The highest superconducting transition temperatures (*T*_c onset_, *T*_cχ′_, and *T*_p_) were also achieved by the Cr-substituted samples for *x* = 0.4 compositions. However, the substitution of Te suppressed the superconductivity of the Tl-1212 phase. *J*_c inter_ (*T*_p_) for all samples was calculated to be in the range of 12–17 A/cm^2^. This work shows that substitution elements with a smaller ionic radius tend to be more favorable in improving the superconducting properties of the Tl-1212 phase.

## 1. Introduction

TlBa_2_CaCu_2_O_7_ and TlSr_2_CaCu_2_O_7_ superconductors are Tl-1212 phases derived from the generic formula Tl_m_A_2_Ca_n−1_Cu_n_O_2n+m+2_, where m = 1, n = 3, and A is either Ba or Sr [1]. It was discovered that TlBa_2_CaCu_2_O_7_ is superconducting at 80 K and could be easily prepared by heating at 900 °C [2]. TlSr_2_CaCu_2_O_7_, in contrast, is often non-superconducting and difficult to prepare in the pure form [3,4,5,6,7]. This is a result of the high average Cu valence (+2.5) and excessive hole carrier doping. Substitution of a higher valence ion decreases the overdoped state and contributes to the superconductivity of TlSr_2_CaCu_2_O_7_. Between +2.25 and +2.35 is the ideal Cu valence for the optimum transition temperature in the Tl-1212 phase [7,8,9]. 

The impact of both Ba and Sr inclusions on the Tl system has been studied. In the study of the Tl-1212 phase, the Tl(Ba,Sr)CaCu_2_O_7_ superconductor was discovered to be superconducting at 90 K. Slow cooling in a nitrogen environment raises *T*_c_ to 94 K [10]. Moreover, a single Tl-2223 phase is easily generated and achieves superconductivity up to 114 K when Ba/Sr = 1 [11]. *T*_c_ of Tl_2_(Ba,Sr)Ca_2_Cu_3_O_10_ was found to be higher than that of Tl_2_Ba_2_Ca_2_Cu_3_O_10_ and Tl_2_Sr_2_Ca_2_Cu_3_O_10_ [12].

Several studies investigated the influence of elemental substitutions on the Tl(Ba, Sr)CaCu_2_O_7_ superconductor. For instance, a post-transition metal (Pb) was found to enhance *T*_c_ of Tl_0.6_Pb_0.4_(Ba,Sr)CaCu_2_O_7_ up to 118 K. The sample also exhibited a single Tl-1212 phase when sintered at 970 °C [13]. In contrast, Ga of post-transition metal was reported to suppress the superconductivity of Tl(Ba,Sr)Ca_1−*x*_Ga*_x_*Cu_2_O_7_ [14]. A transition metal (Ta) was also found to suppress *T*_c_ of (Tl_1−*x*_Ta*_x_*)(Ba,Sr)CaCu_2_O_7_ [15]. There is also a study that identified the effects of non-metal (Se) and metalloid (Te) elements on the superconductivity of Tl_1−*x*_M*_x_*(Ba,Sr)CaCu_2_O_7_ (M = Se or Te) for *x* = 0–0.6 [16]. Se of a non-metal element was reported to enhance the superconducting behavior of Tl(Ba,Sr)CaCu_2_O_7_ in comparison to Te of a metalloid element. The best superconducting behavior resulted in a Se-substituted sample for *x* = 0.3 compositions.

Hence, it is noteworthy to compare the effectiveness of transition (Cr) and post-transition (Bi and Pb) metals versus non-metal (Se) and metalloid (Te) as substitution elements at the Tl site of a Tl(Ba,Sr)CaCu_2_O_7_ superconductor. Some studies mentioned that partial substitution of Bi, Pb, or Cr can help in preserving the Tl-1212 superconducting phase [7,17,18,19]. By doing this, the elements that enhance or suppress the superconducting transition temperature of the Tl(Ba,Sr)CaCu_2_O_7_ superconductor can be investigated.

Tl_1−*x*_X*_x_*(Ba,Sr)CaCu_2_O_7_ superconductors with X = Cr, Bi, Pb, Se, and Te were prepared using a Tl:X ratio of 0.6:0.4. This ratio was chosen by considering the ideal stoichiometry of the Tl-1212 phase, 2.5 − *x*/2. Using *x* = 0.4, the samples were expected to show the best superconducting behavior as the average Cu valence was in the optimum hole-doped state (+2.3). Despite this, Tl(Ba,Sr)CaCu_2_O_7_ and Tl_0.85_Cr_0.15_(Ba,Sr)CaCu_2_O_7_ samples were also prepared for reference purposes. Cr with *x* = 0.15 was used as a reference due to the fact that the highest *T*_c_ in the Tl_1−*x*_M*_x_*Sr_2_CaCu_2_O_7_ superconductor was observed for this composition [20,21].

Hence, it is interesting to study the effects of elemental substitutions at the Tl site of the Tl_1−*x*_X*_x_*(Ba,Sr)CaCu_2_O_7_ superconductor. This work aimed to determine the elements that enhance and suppress the superconducting transition temperature of the Tl_1−*x*_X*_x_*(Ba,Sr)CaCu_2_O_7_ (Tl-1212) phase. Here, the X-ray diffraction (XRD) patterns and scanning electron microscope (SEM) of Tl_1−*x*_X*_x_*(Ba,Sr)CaCu_2_O_7_ for X = Cr, Bi, Pb, Se, and Te are reported together with electrical properties and AC susceptibility results. The relationship between transition temperature and ionic radius of the elements is also discussed.

## 2. Materials and Methods

Preparation of the Tl(Ba,Sr)CaCu_2_O_7_ samples was carried out by solid-state reaction. BaCO_3_, SrCO_3_, CaO, and CuO powders of high purity (≥99.99%) were mixed with a proper ratio and ground using an agate mortar. After thoroughly mixing the powders, they were calcined in air at 900 °C for 48 h with several intermittent grindings. The precursor with the initial formula Tl_1−*x*_X*_x_*(Ba,Sr)CaCu_2_O_7_ had Tl_2_O_3_, Cr_2_O_3_, Bi_2_O_3_, PbO, Se, and Te powders added to it and was well mixed. Pellets of 13 mm in diameter and 2 mm in thickness were pressed from the mixed powders. To make up for the thallium that was lost as a result of the heating process, an extra 10% Tl_2_O_3_ was added. After preheating a tube furnace to 970 °C, the pellets were heated for 4 min in flowing oxygen before the furnace was cooled.

The phase identification and crystal structure determination were examined using X-ray diffraction (XRD) using a Bruker D8 Advanced (Bruker, Billerica, MA, USA) diffractometer equipped with a CuK_α_ source with a wavelength λ = 1.5406 Å. The lattice parameters *a* and *c* for the samples were determined using the least squares method and PTC Mathcad Prime 4.0 software. Each sample’s lattice parameter was measured using at least ten diffraction peaks. The diffraction peaks of Tl(Ba,Sr)CaCu_2_O_7_ (Tl-1212) were indexed by The International Centre for Diffraction Data (ICDD^®^) reference codes 01-083-0677. The percentage of volume fraction was calculated by utilizing the intensity ratios of the diffracted peaks [22]. The field emission scanning electron microscope (FESEM) Merlin Gemini (Zeiss, Oberkochen, Germany) was used to obtain micrograph pictures. Using the Oxford Instrument Analyzer and energy-dispersive X-ray analysis (EDX), the elemental composition was determined. ImageJ was used to compute the grain size and the average grain size of the grains. The grain length was used to estimate the grain size. In order to determine the grain size, up to three length measurements were collected. Meanwhile, the average grain size was measured from the collected data of 100 grains and KaleidaGraph (Synergy Software, Reading, PA, USA) was used to plot the histogram of each sample’s statistical distribution.

The four-point probe approach was used to measure the DC electrical resistance versus temperature. For electrical contact, a silver paste was employed in conjunction with a CTI Cryogenics (Billerica, MA, USA) Model 22 closed-cycle refrigerator. A Lake Shore model 340 temperature controller was used. For the measurements, a constant current source ranging from 1 to 100 mA was used. The AC susceptibility measurements were performed using a Cryo Industry REF-1808-ACS susceptometer (Cryo Industries of America (Manchester, NH, USA)). The frequency employed was 295 Hz while the magnetic field applied was 5 Oe. For the AC susceptibility measurements, the samples were cut into a bar form. To determine the critical current density at peak temperature, *T*_p_ of the imaginary component, and χ″ of the susceptibility, the formula *J*_cinter_(*T*_p_) = *H*/*w* from Bean’s model [23] was employed. *H* denotes the applied magnetic field and *w* represents the cross-sectional dimensions of the bar-shaped sample.

## 3. Results and Discussion

In this section, the results from the XRD patterns, microstructure, electrical resistance, and AC susceptibility measurements are presented.

### 3.1. XRD Patterns

**Figure 1:**
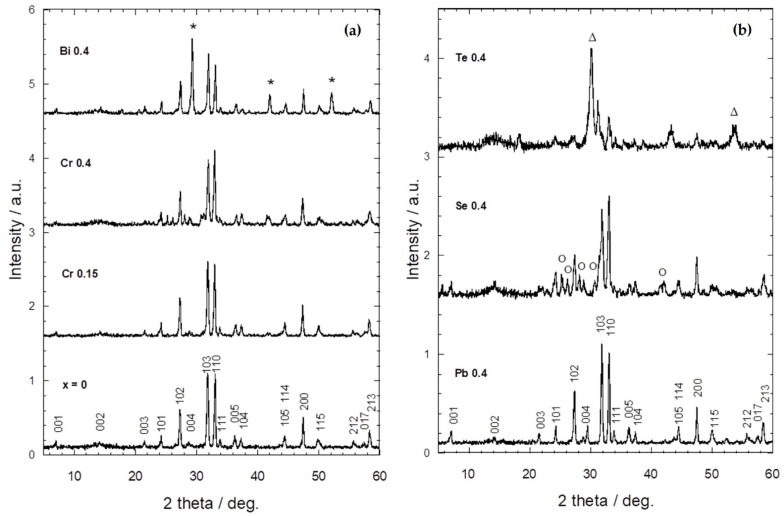
XRD patterns of Tl_1−*x*_X*_x_*(Ba,Sr)CaCu_2_O_7_ with different X substitution. Peaks with (*), (○), and (∆) indicate BaCaBiO^4^, BaSeO_4_, and Ba^2^CaTeO^6^, respectively.

Figure 1a,b show normalized XRD patterns of Tl_1−*x*_X*_x_*(Ba,Sr)CaCu_2_O_7_, respectively. PDF 01-083-0677 was used as the reference phase from the database search of ICDD^®^. A single phase respective to >97% of the Tl-1212 phase was observed in the non- and Cr-substituted (*x* = 0.15) samples. The lattice parameters for the non-substituted sample (*x* = 0) were *a* = 3.8220 and *c* = 12.370 Å. These XRD patterns also show that the Tl-1212 phase was dominant in all samples with a tetragonal unit cell (space group, P4/mmm(123)) (Table 1).

As can be seen from Figure 1, the substitution of Cr, Pb, Bi, Se, and Te elements did not change the intensity peak shift. It is apparent from this result that no structural transition occurred due to the substitution of these elements. However, in Figure 1b, there is a clear trend of a decreasing amplitude of the intensity peak with the substitution of Se and Te. This result indicates that the purity of the Tl-1212 phase was affected by the substitution of non-metal (Se) and metalloid (Te) elements at *x* = 0.4 compositions. There is an existence of minor peaks in the Bi- (Figure 1a), Se- and Te-substituted (Figure 1b) samples for *x* = 0.4. These minor peaks were identified as BaCaBiO_4_ (PDF-00-046-0089), BaSeO_4_ (PDF 01-073-4815), and Ba_2_CaTeO_6_ (PDF 00-0550-1034), respectively. The XRD patterns of Tl_1−*x*_X*_x_*(Ba,Sr)CaCu_2_O_7_ showed different X substitution. Peaks with (*), (○), and (∆) indicate BaCaBiO_4_, BaSeO_4_, and Ba_2_CaTeO_6_, respectively.

Following the variation of the *a* and *c* lattice parameters, the effective ionic radius of the substituted elements with a six coordination number (CN) was considered [24]. The *c* lattice parameter increased as Te was substituted, and this may be due to the ionic radius of Te^4+^ = 0.97 Å being substituted for smaller Tl^3+^ = 0.885 Å. Hence, it is suggested that the effective ionic radius for this work is Te^4+^ = 0.97 Å in comparison to Te^6+^ = 0.56 Å [25] as a result of the increment in the lattice parameters. The decreasing trend of the *c* lattice parameter with the substitution of Cr, Bi, Pb, and Se as in Table 1 is possibly due to their smaller ionic radii being substituted at the Tl site. Considering the ionic radii of Cr, Bi, Pb, and Se for CN = 6 [24], where Cr^3+^ = 0.615 Å [8,26], Bi^3+^ = 1.03 Å [17,27], Pb^4+^ = 0.775 Å [28,29], and Se^4+^ = 0.50 Å [5,16,30], it is recommended that these elements were substituted for a larger Tl site (Tl^1+^ = 1.50 Å and Tl^3+^ = 0.885 Å). These results indicate that there is a significant correlation between the variation of the *a* and *c* lattice parameters with the effective ionic radius of the substituted elements (CN = 6) as mentioned in the previous study [30].

The lattice parameter increased with the substitution of Cr, Pb, and Te elements into the Tl_1−*x*_X*_x_*(Ba,Sr)CaCu_2_O_7_ system. A possible explanation for this is due to the average Cu valence, which may affect the hole-doped state. As the Cr, Pb, and Te ions substituted Tl^1+^/Tl^3+^, the smaller Cu^3+^ converted to the larger Cu^2+^ to fulfil the charge neutrality requirement [31]. Since the lattice parameter primarily depends on the CuO_2_ plane, the conversion of Cu^3+^ into Cu^2+^ caused an expansion of the CuO framework and increased the *a* lattice parameter. Nevertheless, the *a* lattice parameter decreased with the substitution of the Bi and Se elements, which may be due to Bi^3+^ and Se^4+^ substituting Cu^2+^ as their ionic radii are closer to each other with Cu^2+^ = 0.73 Å [24]. However, these assertions need to be supported by a more direct method such as Rietveld refinement and iodometric titration.

### 3.2. SEM Micrograph

**Figure 2:**
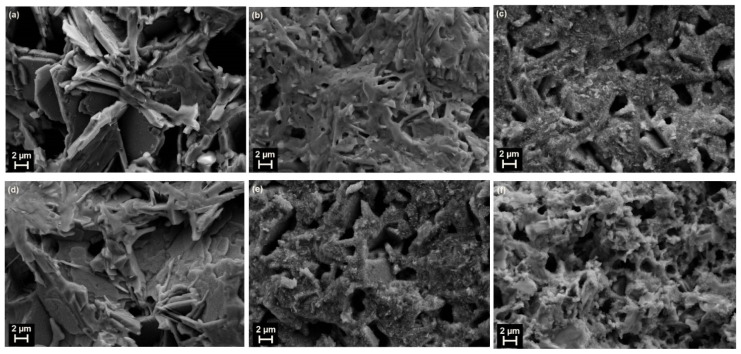
SEM micrographs of Tl_1−*x*_X*_x_*(Ba,Sr)CaCu_2_O_7_ for (**a**) *x* = 0, X(*x* = 0.4) = (**b**) Cr, (**c**) Bi, (**d**) Pb, (**e**) Se, and (**f**) Te.

**Figure 3:**
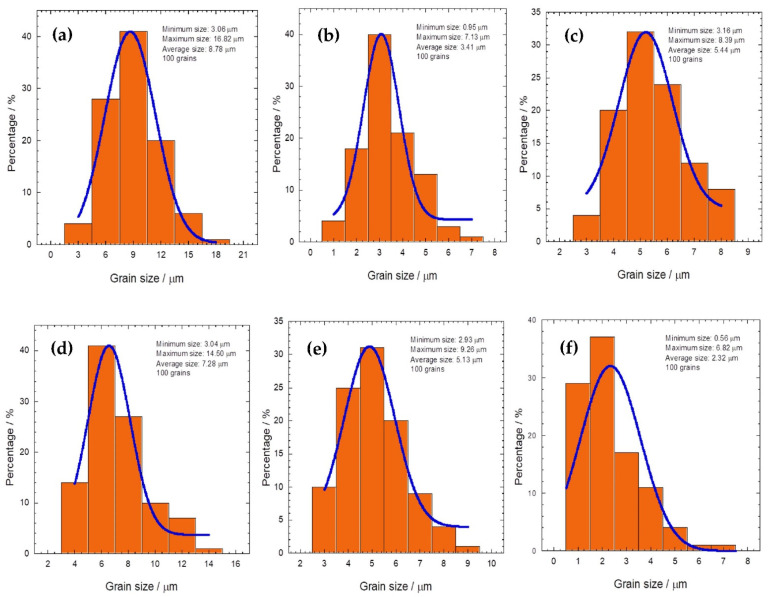
Statistic histogram with a Gaussian distribution fitting curve of the grain size of Tl_1−*x*_X*_x_*(Ba,Sr)CaCu_2_O_7_ for (**a**) *x* = 0, X(*x* = 0.4) = (**b**) Cr, (**c**) Bi, (**d**) Pb, (**e**) Se, and (**f**) Te.

Figure 2 and Figure 3 show the SEM micrographs and statistics histograms with Gaussian distribution fitting curves of the grain size for Tl_1−*x*_X*_x_*(Ba,Sr)CaCu_2_O_7_, respectively. For the purposes of comparison, only the *x* = 0 and 0.4 samples were characterized. The figures were aligned according to (a) *x* = 0, X(*x* = 0.4) = (b) Cr, (c) Bi, (d) Pb, (e) Se, and (f) Te. The average grain size of all samples is shown in Table 1. The average grain size was measured from 100 grains using ImageJ software. The non-substituted sample showed a porous structure with a larger pore size and plate-like structures with well-defined grain boundaries (Figure 2a). The grain size was between 3.058 and 16.817 µm and the average size was 8.78 µm, which is the highest compared to the substituted samples (refer to Figure 3). In contrast, the substituted samples possessed a denser structure with a smaller pore size. The Te-substituted sample (Figure 2f) had the least average grain size (2.32 μm), indicating that Te (metalloid) inhibited grain growth. The average grain size of the transition and post-transition metal-substituted samples (Cr, Bi, and Pb) was around 3.41–7.28 μm (Figure 2b–d), respectively.

The morphology of the non-substituted (*x* = 0) and Pb-substituted (*x* = 0.4) samples, respectively, show a plate-like layered structure with huge voids (Figure 2a,d). Comparatively, the Cr-, Bi-, and Se-substituted samples (Figure 2b,c,e) exhibited a plate-like layered structure with smaller voids, respectively. These defects resulting from the substitution of Cr, Bi, Pb, and Se are expected to work as an efficient pinning center. On the other hand, the grain morphology differed drastically in the Te-substituted sample (Figure 2f). The variation in the microstructure and grain connectivity that affected the transport current density is addressed in the next section, which resulted in the modification of the nature and composition of the intergrain areas [32]. In future work, modern techniques are perhaps necessary for sample preparation such as hot isostatic pressing or spark plasma sintering, which provide nearly zero porosity and a high critical current density.

### 3.3. EDX Analyses

**Figure 4:**
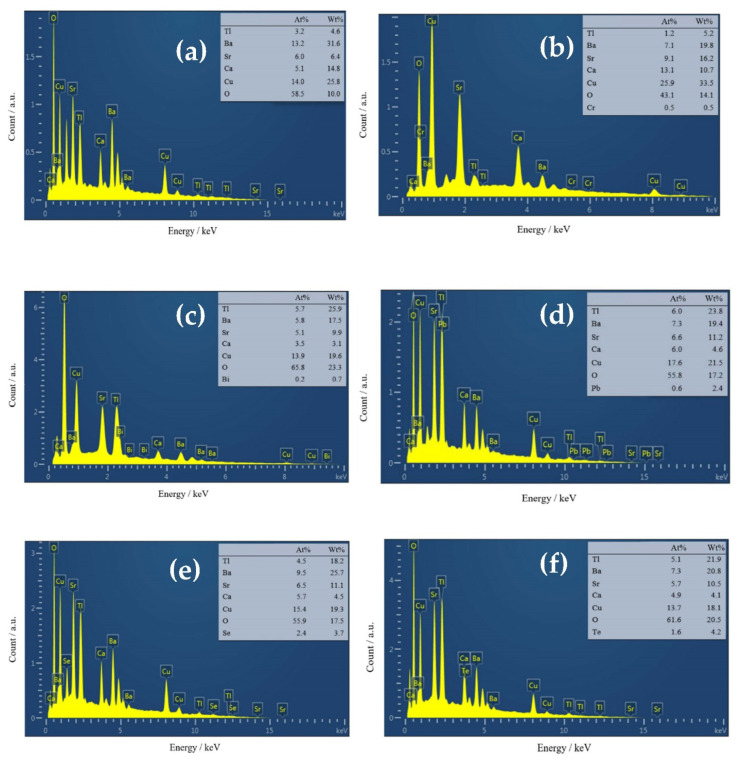
EDX spectra of Tl_1−*x*_X*_x_*(Ba,Sr)CaCu_2_O_7_ for (**a**) *x* = 0, X(*x* = 0.4) = (**b**) Cr, (**c**) Bi, (**d**) Pb, (**e**) Se, and (**f**) Te. Insert shows the atomic and weight percentage of the corresponding elements.

In Figure 4, the EDX spectra of the atomic and weight percentage of the Tl_1−*x*_X*_x_*(Ba,Sr)CaCu_2_O_7_ superconductors for (a) *x* = 0, X(*x* = 0.4) = (b) Cr, (c) Bi, (d) Pb, (e) Se, and (f) Te are shown. In addition to the Tl, Sr, Ca, Cu, and O peaks, the EDX spectra also revealed Cr, Bi, Pb, Se, and Te peaks. The EDX results indicate the chemical composition of Tl-1212 with minor variations due to EDX’s inability to determine light elements such as oxygen and the existence of additional phases such as BaCaBiO_4_, BaSeO_4_, and Ba_2_CaTeO_6_. In Figure 4, the EDX spectra of the atomic and weight percentage of the Tl_1−*x*_X*_x_*(Ba,Sr)CaCu_2_O_7_ superconductors for (a) *x* = 0, X(*x* = 0.4) = (b) Cr, (c) Bi, (d) Pb, (e) Se, and (f) Te are shown. In addition to the Tl, Sr, Ca, Cu, and O peaks, the EDX spectra also revealed Cr, Bi, Pb, Se, and Te peaks. The EDX results indicate the chemical composition of Tl-1212 with minor variations due to EDX’s inability to determine light elements such as oxygen and the existence of additional phases such as BaCaBiO_4_, BaSeO_4_, and Ba_2_CaTeO_6_.

### 3.4. Electrical Resistance

**Figure 5:**
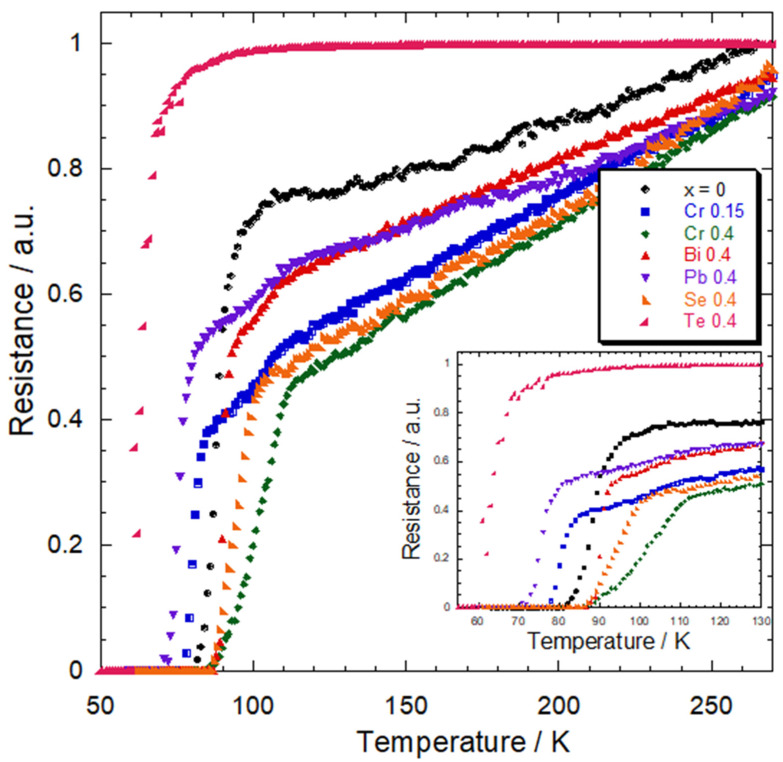
Electrical resistance versus temperature curves of Tl_1−*x*_X*_x_*(Ba,Sr)CaCu_2_O_7_ for X = Cr, Bi, Pb, Se, and Te.

Normalized electrical resistance versus temperature curves of the Tl_1−*x*_X*_x_*(Ba,Sr)CaCu_2_O_7_ samples are shown in Figure 5. All samples show metallic-normal state behavior above the onset transition temperature, *T*_conset_, except for the Te-substituted sample. The Te-substituted sample was observed to exhibit semi-metallic normal state properties. The non-substituted Tl(Ba,Sr)CaCu_2_O_7_ showed *T*_conset_ and a zero transition temperature *T*_czero_ of 96 and 81 K, respectively. Interestingly, all substituted samples were also superconducting, with *T*_conset_ in the range of 77–113 K in which the Te-substituted sample showed the lowest transition temperature for the *x* = 0.4 composition. This indicated that the substitution of the transition metal (Cr), post-transition metals (Bi and Pb), and non-metal (Se) elements improved the doping level and the electrical properties in comparison to the metalloid elements (Te).

However, the Cr- (*x* = 0.15), Bi- (*x* = 0.4), and Pb-substituted (*x* = 0.4) samples exhibited a double transition temperature. This implies that this substitution results in a reduction in the coupling strength among grains [33]. The largest transition width ∆*T*_c_ was observed in the Cr-substituted samples for both *x* = 0.15 and 0.4. This result indicates that Cr increased the variation in the transition temperature of individual superconducting grains. Hence, the doping level and connectivity between grains were improved, which contributed to these samples’ high superconducting transition temperature (Table 1) [34]. Nevertheless, the increase in *T*_conset_ in the Cr-substituted samples could also be due to the improvement of the crystallinity and structure of the samples. It is known that the resistivity in the normal state of HTS depends on the porosity and grain boundary scattering in the samples. In this study, Cr substitution may alter the electron mean free time due to the greater number of disorders in the structure. This is independent of the homogeneity and oxygen content in the samples since all the samples were prepared under identical conditions. However, such a study needs to be carried out in detail with other calculations and experiments.

It is interesting to note that the transition temperature with *T*_conset_ ≥ 100 K was observed in the Cr- and Se-substituted samples. This may be explained in terms of the effects of the ionic radius and valence states of elements. Both Cr and Se elements have a smaller ionic radius and exist in a multivalence state. The size of the ionic radius for Cr and Se was within the range of the ionic radius of the substitution site (Tl and Cu). Despite having a smaller ionic radius, a recent study revealed that Cr and Se increased *T*_c_ of the Tl-1212 phase [30]. In line with this point, our results implied that Cr^3+^ and Se^4+^ with smaller ionic radii induced a high superconducting transition temperature. Regarding the valence state of elements, Tl^1+^/Tl^3+^, Cr^2+^/Cr^3+^/Cr^4+^/Cr^5+^/Cr^6+^, Se^4+^/Se^6+^, Ba^2+^, Sr^2+^, Ca^2+^, and Cu^2+^/Cu^3+^ are the possible valence of the ions in the Tl_1−*x*_X*_x_*(Ba,Sr)CaCu_2_O_7_ superconductor with X = Cr and Se, respectively. Tl, Cr, and Se were found to favor a single valence state in the Tl-1212 phase with Tl^3+^ [35], Cr^3+^ [8,26], and Se^4+^ [5,16,30]. Interestingly, the role of Cr in the Tl-1212 phase is highly distinctive and merits specific consideration. The Jahn–Teller effect contributed to the mechanism of increased *T*_c_ in samples with Cr substitution. Cr can occupy the Tl^3+^ site in Tl_1−*x*_X*_x_*(Ba,Sr)CaCu_2_O_7_ due to the Jahn–Teller nature of Cu, which permits a great degree of flexibility in the Cu–O apical distance [31].

Our results show that the substitution of trivalent (+3) and tetravalent (+4) elements increased the superconductivity. These results are similar to a recent study, which reported that elements with higher valences enhanced the transition temperature and are effective in stabilizing the Tl-1212 phase [30]. Moreover, this study aimed to compare the efficacy of multivalent elements with the same stoichiometric ratio on the superconductivity of the Tl-1212 phase. Thus, the optimal carrier concentration is not desirable. Moreover, the optimal carrier concentration in cuprates does not necessarily result in a superconducting phase [6].

### 3.5. AC Susceptibility

**Figure 6:**
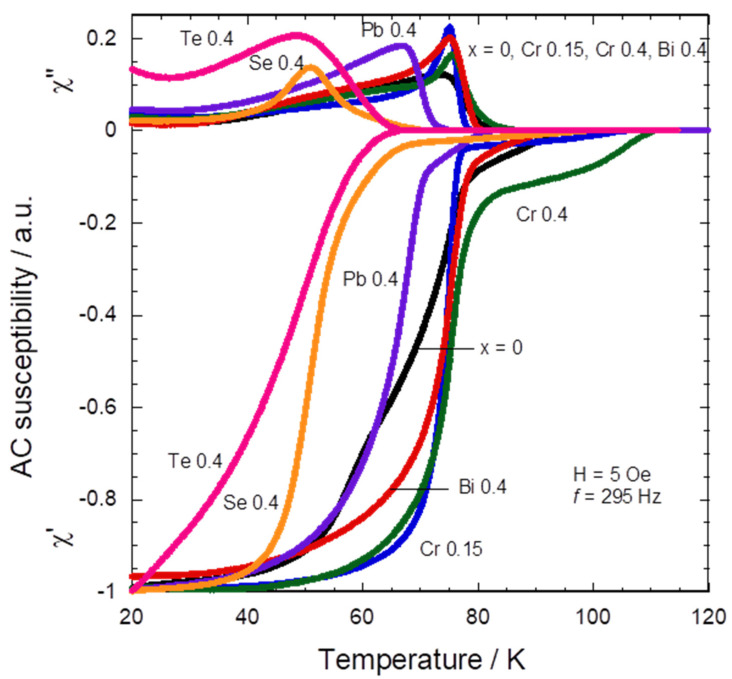
AC susceptibility (χ = χ′ + iχ″) versus temperature of Tl_1−*x*_X*_x_*(Ba,Sr)CaCu_2_O_7_ for X = Cr, Bi, Pb, Se, and Te.

Figure 6 shows the AC susceptibility measurement of the Tl_1−*x*_X*_x_*(Ba,Sr)CaCu_2_O_7_. This is a non-destructive tool used to measure the bulk superconductivity by identifying the inter-grain characteristics [33]. The real part χ′ of the AC susceptibility measurement (χ = χ′ + *i*χ″) represents diamagnetic behavior whereas the imaginary part χ″ illustrates the nature of the flux pinning strength and the connection between the grain. The susceptibility transition temperature *T*_cχ′_ generated by diamagnetic shielding is shown by a sudden transition in the χ′ part. *T*_cχ′_ is also the transition temperature at which bulk superconductivity begins. Two peaks can be noticed in the χ″ part of the susceptibility, which was caused by the AC losses. At higher temperatures, the first loss peak (intrinsic losses) may be visible, exhibiting an intragranular current density (*J*_cintra_). In contrast, the area with the lower temperature displayed the second loss peak (coupling losses) as an indication of the intergranular current density (*J*_cinter_) and grain coupling qualities. Both χ″ loss peaks are affected by the applied magnetic field.

The absence of an intragranular loss peak in all samples may be a result of the low magnetic field, *H*_ac_, that was applied (Figure 6). This demonstrated that the administered *H*_ac_ was insufficient to penetrate the grains. Nevertheless, *H*_ac_ was adequate for penetrating within the grains and showed the presence of an intergranular loss peak (*T*_p_) [33]. At a given magnitude of the applied magnetic field (5 Oe), the degree of shifting and breadth of the intergranular loss peak may be used to determine the strength of flux pinning. In addition, Bean’s model can be used to calculate *J*_cinter_ at *T*_p_ (*J*_cinter_ (*T*_p_)) since the magnitude of *H*_ac_ is equal to the flux that has penetrated *T*_p_ [23].

Our result showed that *T*_cχ’_ for Tl_1−*x*_X*_x_*(Ba,Sr)CaCu_2_O_7_ is in the range of 62–111 K. The highest *T*_cχ′_ was recorded by both Cr-substituted samples for *x* = 0.15 and 0.4, respectively (107 and 111 K). This result implies that Cr sped up the flux penetration and pinned the vortex motions through diamagnetic shielding. The double transition of *T*_cχ′_ in the substitution of Cr, Bi, Pb, and Se reflects the inhomogeneities of samples, showing that these elements were expected to efficiently work as pinning centers [33]. Whereas *T*_p_ is the maximum temperature in which full flux penetration occurred in the sample. A single peak associated with the loss in the sample at a temperature of *T*_p_ was observed for all samples. This loss peak shifts to a higher temperature with the substitution of Cr and Bi, which implies that the flux pinning force is increased with these element substitutions. The Cr- and Bi-substituted samples also narrowed the curves and increased *T*_p_ in comparison to the non-substituted sample. This indicates that the intergranular coupling strength and connection are improved with Cr and Bi substitution, respectively. *J*_cinter_ (*T*_p_) for the Tl_1−*x*_X*_x_*(Ba,Sr)CaCu_2_O_7_ superconductor was found to be in the range of 12–17 A cm^−2^ with *T*_p_ ~ 0.9*T*_c_.

Our results on the AC susceptibility measurement showed that *T*_cχ’_ and *T*_p_ are the highest in the Cr-substituted sample for the *x* = 0.4 composition (111 and 76 K). These results implied that the susceptibility transition exhibited better intergranular characteristics with Cr substitution [32]. Cr also improved the morphology of the Tl-1212 phase by decreasing the size of the voids (Figure 2b), in comparison to the non-substituted sample (Figure 2a). Reducing the voids increased intergranular coupling and increased the transition temperature, which was also found in another Cr-substituted Tl-1212 [21]. Thus, it is suggested that Cr substitution creates efficient pinning centers for the Tl-1212 phase in comparison to Bi, Pb, Se, and Te.

Unlike prior studies of the Tl_1−*x*_Cr*_x_*Sr_2_CaCu_2_O_7_ superconductor [8,21,26], our Tl_0.85_Cr_0.4_(Ba,Sr)CaCu_2_O_7_ sample demonstrated a higher transition temperature (*T*_conset_ = 113 K). In addition, comparable effects of a single Tl-1212 phase were seen in close materials of the Tl(Ba,Sr)CaCu_2_O_7_ superconductor following the same sample preparation [13,14,15]. In recent studies investigating the effects of similar substitutions, Cr demonstrated superior superconducting properties in terms of the superconducting transition temperature compared to Ga and Ta. 

## 4. Conclusions

The effects of elemental substitutions at the Tl site of the Tl_1−*x*_X*_x_*(Ba, Sr)CaCu_2_O_7_ superconductor were studied. The Cr-substituted samples showed the highest *T*_conset_ for *x* = 0.4 although the non- and Cr-substituted samples (*x* = 0.15) exhibited a single Tl-1212 phase. The Cr-substituted sample also reduced the voids’ size of the Tl-1212 phase morphology, which resulted in an improved intergranular characteristic. Both *T*_cχ’_ and *T*_p_ were found to be the highest in the AC susceptibility measurement of the Cr-substituted samples. In contrast, the Te-substituted sample suppressed the superconductivity of the Tl-1212 phase. Thus, substitution at the Tl site by Cr (Cr^3+^ = 0.615 Å) exhibited better superconducting behavior in the Tl-1212 phase than Te (Te^4+^ = 0.97 Å) in preserving the Tl-1212 phase, enhancing the superconducting transition temperature (*T*_conset_, *T*_cχ′_, and *T*_p_) and being effective enough to act as an artificial pinning center for the same stoichiometric ratio with *x* = 0.4. This work also showed that a substituted element with a smaller size ionic radius exhibits better superconducting behavior in the Tl-1212 phase. This study suggests that the ionic radius of the substituted elements must be relatively small or within the range of the substitution site to facilitate the superconducting behavior of the Tl-1212 phase and to increase the transition temperature.

## Figures and Tables

**Table 1:** *T*_conset_, *T*_czero_, ∆*T*_c_, *T*_cχ′_, *T*_p_, *J*_c inter_(*T*_p_), average grain sizes, lattice parameters, unit volume cell, and volume fraction of Tl-1212 phase for Tl_1−*x*_X*_x_*(Ba,Sr)CaCu_2_O_7_ with X = Cr, Bi, Pb, Se, and Te.

Tl_1−*x*_X*_x_*(Ba,Sr)CaCu_2_O	*T*_conset_ (K)	*T*_c zero_ (K)	Δ*T*_c_ (K)	*T*_cχ′_ (K)	*T*_p_ (K)	*J*_c_ (*T*_p_) (Acm^−2^)	Average Grain Size ± SD/μm	*a* (Å)	*c* (Å)	*V* (Å^3^)	Tl-1212 Phase (%)
*x* = 0	96	81	15	95	74	17	9 ± 3	3.8220	12.370	180.70	>97
X = Cr (*x* = 0.15)	106	77	29	107	75	12	-	3.8320	12.304	180.67	>97
X = Cr (*x* = 0.4)	113	85	28	111	76	14	3 ± 1	3.8270	12.289	179.98	83
X = Bi (*x* = 0.4)	95	87	8	92	75	15	5 ± 1	3.8170	12.282	178.94	68
X = Pb (*x* = 0.4)	82	70	8	83	67	12	7 ± 2	3.8250	12.327	180.35	95
X = Se (*x* = 0.4)	100	85	15	100	51	17	5 ± 1	3.8190	12.314	179.99	78
X = Te (*x* = 0.4)	77	60	17	62	48	16	2 ± 1	3.8240	12.394	181.21	51

Note: SD is the standard deviation.

## Data Availability

Data will be made available upon reasonable request.
